# Lucy Series, *Deprivation of Liberty in the Shadows of the Institution*

**DOI:** 10.1093/medlaw/fwac029

**Published:** 2022-08-10

**Authors:** Ruby Reed-Berendt

**Affiliations:** School of Law, University of Edinburgh, Scotland

In the UK’s current social care model, individuals with intellectual or cognitive disabilities (eg dementia, autism, or other developmental conditions) who are deemed to lack mental capacity often see decisions made by others regarding their residence, care, and other broad elements of their daily life. But if they are happy with this and living lives of normalcy in a home, should we consider them to be deprived of their liberty for the purposes of article 5 of the European Convention on Human Rights (ECHR)? Currently, the legal answer to this question is yes, because a ‘gilded cage is still a cage’.^1^ Such was the reasoning of Lady Hale in the leading *Cheshire West* judgment. This introduced an ‘acid test’ for determining whether an individual was deprived of their liberty which, rather than relying on an institutional location, instead focuses on whether an individual is under continuous supervision and control, and unable to leave.^2^ The implications of the judgment for the provision of social care across the UK are also profound, and the case has contributed to the replacement of England and Wales’ Deprivation of Liberty Safeguards (DoLS) with the Liberty Protection Safeguards (LPS) (at the time of writing, the timescale for LPS implementation remains unclear).

Against the backdrop of this shifting legal context, Series’ thought-provoking book aims to make sense of the modern legal and policy landscape through the lens what she terms ‘social care detention’. To do so, she uses critical genealogy to explore how the current approach and characterisation of the issue of ‘deprivation of liberty’ is deeply rooted in (and to some extent, reproduces) historical, carceral, responses to mental disability. This provides an important contribution to the ongoing academic discourse on deprivation of liberty specifically, but also is of interest for its critical analysis of law’s approach to disability and mental health. Throughout the book’s eleven chapters, Series draws out key themes concerning the paradoxes of social care detention, power and domination in the system, and the increasingly untenable distinction between ‘community’ and ‘institutional’ spaces of care. Her approach naturally means that large portions of the book focus on the history behind the current system, however, this is by no means to its detriment. Instead, it allows Series to pose a number of important questions to the reader, namely: Has our focus on legalistic solutions to social care detention and the ‘liberty’ problem meant we have lost sight of what issues we are trying to solve? And can we imagine new models or paradigms which allow us to move beyond our carceral past?

Following an important note on terminology (which is considerate of the legacy of stigma attached to labels in mental health, and the challenges of appropriate descriptors) and brief introduction, Series sets the foundations for the rest of her discussion by exploring the concept of social care detention. In chapter 2, she distinguishes social care detention and its framing under the Mental Capacity Act 2005 (MCA) with the more traditional detention powers of the Mental Health Act 1983 (as amended in 2007) (MHA). A detailed picture is painted of how social care detention targets different populations, applies across a semi-permanent timeframe, and operates different legal technologies focused on empowerment and consideration of wishes and feelings. This mode of detention lies beyond the more familiar institutional routes of the MHA. Series then convincingly argues that despite these calls to empowerment and rejection of risk management, social care detention operates similar coercive powers in the name of addressing vulnerability. She depicts the MCA as fundamentally providing a defence to those who seek to treat the patient without consent (section 5 provides a general defence against liability as long as the person doing ‘the act’ reasonably believes the individual lacks capacity and it is in their best interests), but containing far fewer substantive and procedural safeguards than the MHA. This not only provides a frame for the discussion to come, but also gives important insight into the complexity of social care detention and it’s at times contradictory rationalities.

Chapters 3 and 4 provide a detailed analysis of the evolution of social care detention from its roots in the eighteenth-century ‘lunacy laws’ to the twentieth-century move to ‘the community’. Chapter 3 explores the development of the ‘law of institutions’, which Series characterises as (i) the law which regulates detention itself and (ii) the law which regulates the setting in which the detention takes place. Starting from the Madhouses Act 1774, she traces the legal developments which led to the proliferation of ‘asylums’ during the nineteenth century, and how medical control and authority were cemented through this. Having considered what resistance there was to this, by families and the Commission regulating the system, she then aptly demonstrates how the law moved to distinguish ‘mental disorder’ and mental disability. Noting the uneasy space occupied by mental disabilities and their perceived ‘untreatability’ in the institutional system, Series unearths how these individuals came to be managed under a distinct legal framework (the Mental Deficiency Act 1913), which provided a separate and overstretched institutional model for this population.

Picking up from the climax of institutional provision, Chapter 4 turns to its decline. Here, Series first focuses on how narratives were constructed to support the community as the proper place for managing mental disability. However, institutional practices permeated the reforms, even if such practices were less visible in a post-carceral context. The paradoxes arising from this context come across strongly in this chapter, as Series clearly demonstrates how notions of independence advanced by activists against institutional care have justified coercive interventions, as well as how the ideal of a ‘least restrictive’ intervention problematically suggests that some form of restriction will be warranted. This provokes the reader to reflect on how the history of institutional care continues to shape the modern social care context. She then provides an overview of the two ‘waves’ of deinstitutionalisation, the move from medical care in hospitals to social care in care homes, and then the move to supported living in the community. Through this discussion, Series highlights the importance and constraints of service provision, pointing to how the changes were driven by aims to increase choice for the person. However, such choices appear to relate to ‘who’ provides support, and may be constrained if support is not funded and therefore not available. The chapter ends with a discussion of the family and their uneasy place in post-carceral care.

Chapter 5 moves to consider the interaction between social care detention and human rights frameworks, namely the ECHR and UN Convention on the Rights of Persons with Disabilities (CRPD). After considering how disabled people came to be recognised as international rights-bearers, Series explores how social care detention became an issue under article 5 ECHR, and the evolving case law in the European Court of Human Rights. She then demonstrates how article 5 compliance has led to the resurgence of the law of institutions, with responses rooted in creating a legal framework with safeguards on social care detention, and regulatory frameworks to monitor it. The second part of the chapter then considers the ‘new paradigm’ CRPD framework, which seeks to the abolish legal frameworks based on disability and asserts the enjoyment of rights for persons with disabilities on an equal basis with others. She highlights how, despite the CRPD’s rejection of the notion of ‘mental incapacity’ and aligned restrictions on liberty, there has been little recognition that social detention in a community setting can in fact result in infringements on liberty. An important point is made here about the need for CRPD advocates and their so-called ‘abolitionist’ perspective to engage with institutional practices which may endure in the post-institutional landscape and what liberty ought to mean in their non-discrimination paradigm.

Chapter 6 focuses on the decision spaces of our post-carceral context and in particular the dichotomy between home and the institution. Series argues that central to this ideology is that individual’s minor decisions and ‘little choices’ are key in promoting their wellbeing and allowing their sense of self and identity to develop. With the institution depicting an environment where everything is controlled, the home, by contrast, is a space where the individual has choice and control, as well as being able to develop in a social world. Series explores the dimensions of the home by illustrating three elements of ‘home’, first its territorial dimension as a place of privacy and control, second its connection with developing self-identity, and third its social and cultural dimension, which facilitates connections with family and inclusion in a wider community. This is juxtaposed with how the institution imposes its own wishes and goals on individuals, control physical spaces and remove individuals from their community locus. Yet, as Series highlights, if the home is a space where individual does not make decisions, a space of caregiving is created which is closer to the institution, thus dissolving an individual’s sense of home and blurs the boundary between the two. Drawing on the concept of liminal spaces, Series demonstrates how social care detention has created various spaces that look like homes but have cultural aspects or dynamics of the institution, and conversely there are institutions where residents have found a sense of home. She, therefore, suggests that home could instead be seen as a ‘decision space’ which gives rise to possibilities for decision-making on a macro-level (where to live) and micro-level (everyday small decisions on how to live). She leaves open the question of whether, in our modern economic context, law would be able to deliver our aim of securing true homes for adults who were previously institutionalised.

After laying this foundation, the second half of the book concentrates on the DoLS and *Cheshire West* more explicitly, from nascent beginnings, through to the case’s aftermath and beyond. Beginning with the Mental Health Act 1959, Chapter 7 considers the advent of informal treatment (as opposed to compulsory admission) and the non-volitional patient who was incapable of voluntary treatment but did not actively object. In this chapter, Series highlights how this idea of a patient who is not actively objecting endures through the MCA, which embeds concepts of informality, MHA’s distinction between objecting and resisting patients and those who can be admitted informally. The other section of the chapter is devoted to the regulatory landscape of post-carceral care. Series demonstrates here the challenges of regulating a space where the boundaries between institutional and domestic arrangements are far less obvious, focusing in particular on residential care provision and supported living services. She provides an insightful picture of the role and limitations of the Care Quality Commission (CQC) in the modern market, noting their ability to consider micro-level decisions which provide some choice and control but not wider macro-level aspects. The chapter ends by introducing the *Bournewood* case,^3^ which established informal admission could nonetheless deprive an individual of their liberty, and created in its wake the formal DoLS system. This sets up the discussion to come as the courts are forced to engage with the issue of deprivation of liberty, its contours and limitations.

Series turns in Chapter 8 to *Cheshire West* itself. She begins by summarising the facts of the three individuals at its centre, MEG, MIG, and P, before providing details of the courts’ approach to deprivation of liberty before *Cheshire West.* She distils from prior judicial reasoning a wish to maintain the boundary between the institution and the community through their definition of what constitutes a deprivation of liberty. Series highlights in particular how ‘home’ and ‘family’ provision (as a site of freedom in judicial discussion), the ‘normality’ of the arrangements, the benevolent reasons for any restrictions, and lack of objection were used by judges in finding there was no deprivation of liberty. Through this discussion, Series emphasises how their approach legitimised and normalised control being exercised in the family home, as well as embedded assumptions about what a normal life would be for a disabled adult. She then discusses *Cheshire West* itself, how the arguments previously made were overturned, and the consequence that deprivation of liberty may take place in private homes. The chapter concludes by examining the response to, and backlash against, *Cheshire West*, in judicial, academic, and activist spheres. This sets the stage for chapter 9, which discusses what came next.

Series starts her exploration of the aftermath of *Cheshire West* with the immediate impact on the number of DoLS applications made, and how the case essentially forced the government to rethink their approach in the face of a broken system. She gives an overview of the LPS and the process of reform, the key features of the new safeguards, and subsequent litigation which considered medical treatment and the position of children. In Chapter 9, Series begins to question what the outcome of this reform actually is. She asks what the LPS are supposed to be safeguards against, given they focus on ‘arrangements’ for care (and capacity to consent to them), rather than what care is given and where. Are they an independent check on the substitute decisions or simply the arrangements that enable them? What are we trying to achieve by saying even if someone is happy, they have been deprived of their liberty? And why are we turning to law? These questions become the focus of the final two chapters of the book.

Beginning the turn to the implications of the new legal technologies of social care detention, Chapter 10 engages with its notions and rationalities of protection and vulnerability, with a focus on power and domination. In one of the most interesting parts of the book, Series begins by noting that *Cheshire West’*s implication is that social care detention may be designed to alleviate vulnerability, but its mechanisms can themselves generate vulnerability. She then notes that ECHR compliance does not seek to prevent deprivation of liberty but ensures it reflects social norms and does not engender the unrestrained use of discretionary power, or, on a civic republican view, domination. Series goes on to depict social care as a landscape of domination on a macro and micro level, which permits the restriction on day-to-day choices as well as over where individuals live. Series suggests that the modern system embodies ‘care-professional legalism’,^4^ in that it consists of a series of trained professionals who interpret the MCA, approve DoLS, and influence individuals’ care arrangements, with their voice apparently absent in these discussions. She closes the chapter by considering alternative strategies such as substituted consent or a CRPD-style model that might offer any assistance in tackling domination, noting that even this may struggle to correct the challenges of our current model.

The book concludes by considering how social care might move beyond the law of institutions. Series argues that the safeguards we have now built into social care detention are framed as being *for* liberty and *against* the reality of restrictive practices in community settings. They are also underpinned by a perceived need to use legal controls to mitigate excessive forms of power. However, as she reminds the reader, social care detention embodies a form of significant legalism which stretches across vast communities in any space, with a different cast of professionals who bring a different framing to living arrangements. It is also a system characterised by restraints on funding and heading towards a crisis of provision. Exacerbated by the pandemic, this is not an issue that legal technologies can solve. From this, Series argues that to move beyond institutional models, we need to reconceptualise what issues we are trying to solve and what ideals we are looking to promote. She proposes a need to look at the ‘goal’ as trying to provide decision spaces for individuals within the system, providing them with support to express their views and live in accordance with those views, and promoting the availability of these spaces. This, Series suggests, would allow consideration of which decisions should be subject to scrutiny by the state, why individuals require supervision, and in what circumstances their choices should be constrained.

Whilst we continue to await the implementation of the LPS, Series’ book provides an important perspective on the rationalities, legal technologies, and power relations embedded in the current framework. Based on Series’ critique, it does not appear that the LPS will offer a drastically different future and will continue to provide a response to deprivations of liberty rooted in law. In the context of the upcoming reform of the MHA, which seems set to have profound implications for the treatment and care arrangements for individuals with autism and learning disability, and may indeed see increasing use of the LPS,^5^ the issues raised by Series are of importance not only for medico-legal scholarship, but also for policymakers and activists. It encourages a reconsideration of the goals, and appropriate limits, of compulsion, and what law in mental health and capacity should seek to achieve. Aside from providing a cautionary note about repeating the patterns of the past, Series’ book offers an alternative future to reformers, where individuals subject to social care detention might be afforded ‘decision spaces’ and provided with the opportunity to make their residence, wherever it may be, resemble a home.

## Footnotes

1
*P (by his litigation friend the Official Solicitor) v Cheshire West and Chester Council and another; P and Q (by their litigation friend, the Official Solicitor) v Surrey County Council* [2014] UKSC 19, [46].

2 ibid [49].

3
*R v Bournewood Community and Mental Health NHS Trust ex parte L* [1998] UKHL 24; *HL v United Kingdom* 45508/99 [2004] ECHR 47.

4 L Series, *Deprivation of Liberty in the Shadows of the Insitution,* (Bristol University Press 2022), pp 218–19.

5 For a discussion, see Rosie Harding, *Initial Thoughts on the Draft Mental Health Bill: Autism and Learning Disability Provisions* (29 June 2022) <https://legalcapacity.org.uk/crpd/initial-thoughts-on-the-draft-mental-health-bill-autism-and-learning-disability-provisions/> accessed 26 July 2022.

